# Ion Prostate Irradiation (IPI) – a pilot study to establish the safety and feasibility of primary hypofractionated irradiation of the prostate with protons and carbon ions in a raster scan technique

**DOI:** 10.1186/1471-2407-14-202

**Published:** 2014-03-19

**Authors:** Gregor Habl, Gencay Hatiboglu, Lutz Edler, Matthias Uhl, Sonja Krause, Matthias Roethke, Heinz P Schlemmer, Boris Hadaschik, Juergen Debus, Klaus Herfarth

**Affiliations:** 1 Department of Radiation Oncology, University of Heidelberg Medical Center, Heidelberg, Germany; 2 Department of Urology, University of Heidelberg Medical Center, Heidelberg, Germany; 3 Department of biostatistics, DKFZ (german cancer research center) of Heidelberg, Heidelberg, Germany; 4 Department of radiology, DKFZ (german cancer research center) of Heidelberg, Heidelberg, Germany

## Abstract

**Background:**

Due to physical characteristics, ions like protons or carbon ions can administer the dose to the target volume more efficiently than photons since the dose can be lowered at the surrounding normal tissue. Radiation biological considerations are based on the assumption that the α/β value for prostate cancer cells is 1.5 Gy, so that a biologically more effective dose could be administered due to hypofractionation without increasing risks of late effects of bladder (α/β = 4.0) and rectum (α/β = 3.9).

**Methods/Design:**

The IPI study is a prospective randomized phase II study exploring the safety and feasibility of primary hypofractionated irradiation of the prostate with protons and carbon ions in a raster scan technique. The study is designed to enroll 92 patients with localized prostate cancer. Primary aim is the assessment of the safety and feasibility of the study treatment on the basis of incidence grade III and IV NCI-CTC-AE (v. 4.02) toxicity and/or the dropout of the patient from the planned therapy due to any reason. Secondary endpoints are PSA-progression free survival (PSA-PFS), overall survival (OS) and quality-of-life (QoL).

**Discussion:**

This pilot study aims at the evaluation of the safety and feasibility of hypofractionated irradiation of the prostate with protons and carbon ions in prostate cancer patients in an active beam technique. Additionally, the safety results will be compared with Japanese results recently published for carbon ion irradiation. Due to the missing data of protons in this hypofractionated scheme, an in depth evaluation of the toxicity will be created to gain basic data for a following comparison study with carbon ion irradiation.

**Trial registration:**

Clinical Trial Identifier: NCT01641185 (clinicaltrials.gov)

## Background

The success of irradiation in patients with localized prostate cancer correlates with the administered dose [1-5]. This is well known for patients with an intermediate risk profile and could also be found recently for patients with a low risk profile (Gleason score < 7; PSA < 10 ng/ml) [6]. Limitations for using higher doses are due to an increase of complication rates in particular to the rectum (bleedings, fistula, ulcer), urethra (stenosis) and bladder (chronic cystitis).

The rate of adverse effects is not only dependent on the dose but also on the radiation technique used. An earlier randomized study found that the rectum toxicity was lowered significantly when applying 3D-CT based radiation planning compared with simulator based planning [7]. In a dose escalating study at the MD Anderson, Pollack and co-workers found a volume dependency of the rectum toxicity (rectum volume irradiated with > 70 Gy, toxicity of grade II or higher after 5 years was 13% or 51% by ≤25% or >25% rectum volume, respectively [8]. Due to the use of intensity modulated radiotherapy it is possible to increase the doses up to 76–81 Gy whereas the adverse effects could, in comparison to 3D conformal radiotherapy, be lowered significantly using the same dosage reaching the level obtained with radiation series giving a total dose of 64–70 Gy. This monocentric historical comparison showed also a significantly higher cure rate [5].

Radiation biological considerations act on the assumption that the α/β value (tissue specific constant specifying the tissues’ sensitivity for the possibility of developing a late toxicity if single doses are increased) of the prostate cancer cells is low [9]. Since the assumed α/β value of 1.5 Gy for prostate cancer cells is clearly below the α/β values of bladder (α/β value 4.0) and rectum (α/β value 3.9), a radiation biological more effective dose could be administered due to increased single doses without increasing risks of late adverse effects. At the same time, the overall treatment time is shortened. This radiation biological hypothesis was confirmed in a study of 770 patients receiving a total dose of 70 Gy in 28 fractions, single doses of 2.5 Gy where biochemical recurrence free survival was 83% after 5 years for all risk groups, 95% for the low risk group with only 2% acute and late grade 3 or higher toxicity [2].

Due to physical characteristics, ions like protons or carbon ions can administer the dose to the target volume more efficiently than photons since the dose can be lowered to the surrounding normal tissue. However, parts of the risk organs remain in the target volume: base of bladder, urethra and the facing wall of the rectum. The tolerance dose of the urethra, which is in the center of the target volume, is >85 Gy. A prospective study with a fraction scheme of 48 × 1.8 Gy = 86.4 Gy reported the appearance of urethral strictures in <3% of the patients [10]. For both, the bladder and rectum, studies found a high volume effect. The TD 5/5 for radiation of 2/3 of the bladder is 80 Gy, whereas it is 65 Gy for radiation of the whole bladder [11]. For the rectum, a high volume effect is known as well [12]. With ion radiation, it is possible to spare especially the posterior rectum wall in comparison to photon radiation, so potentially receiving also a more efficient regeneration of the anterior rectum wall. For proton irradiation, we know the data of the retrospective analysis of Loma Linda [13], as well as the randomized study of Boston, in which proton boost to a TD of 79.2 Gy vs. 70.2 Gy was used after a radiation of photons. The authors showed a significant advantage for high doses with an acceptable occurrence of adverse effects (2% acute and chronic toxicity < of CTC grade 3 or higher) [6].

Due to its mass, carbon ions have a higher biological efficacy than protons with comparable dose profile. Several trials using carbon ions in a dose escalating manner were performed by the group of researchers in Chiba/Japan. A total of 176 patients were treated with a dose of 20 × 3.3 GyE. The 5-year biochemical recurrence free survival rate was up to 83.2% for all patients and 100% for patients with low risk prostate cancer. No late toxicity of CTC grade 3 or higher was found [3]. However, the region of the anterior rectum wall was only strained to 50-90% of the dose.

All published studies of irradiation of the prostate with ions use a passive beam modulation. Currently developed is ion radiation with active beam modulation and raster scan technique [14]. The target volume is radiated point-by-point in contrast to the radiation in layers in passive beam modulation. The advantage of this method is the lower production of neutrons, and as such the lower risk of the occurrence of secondary malignancies. Despite the more exact dose application, the passive beam modulation has no advantage in developing secondary cancer compared to IMRT irradiation [1].

In an initial study in our department, we tested the feasibility and safety of the active beam modulation of carbon ion irradiation of the prostate. We used a carbon ion boost of 6 × 3 GyE in combination with an IMRT photon radiation of 30 × 2 Gy. An interim analysis showed a good response rate and no increased toxicity [15]. On the other hand, we could not identify data for hypofractionated irradiation of the prostate with protons so far.

To spare the rectum additionally and to consider the movement of the prostate during the irradiation [16], an absorbable gel can be injected between the rectum and the prostate. In a prospective multi-center trial, we could show that the rectum dose could be significantly lowered using this gel that established a stable gap of 7–10 mm between rectum and prostate for approximately 6 months [17,18].

The planned IPI study is a prospective randomized phase II study exploring the safety and feasibility of primary hypofractionated irradiation of the prostate with protons and carbon ions in a raster scan technique and is planned to enroll 92 patients with localized prostate cancer. Primary endpoint is the portion of patients to whom the study treatment can be safely applied (i.e. without grade III and IV NCI-CTC-AE (v. 4.02) toxicity) and/or without dropout from the planned therapy due to any reason (rate of safety and feasibility). Secondary endpoints are PSA-progression free survival (PSA-PF, overall survival (OS) and quality-of-life (QoL).

## Methods/Design

### Trial organization/coordination

The IPI study is designed as an open-label, prospective, single-center, randomized two-armed (proton vs. carbon ion irradiation) pilot study evaluating the safety and feasibility of hypofractionated irradiation of the prostate with protons and carbon ions in prostate cancer patients in an active beam technique designed by the study initiators of the Department of Radiation Oncology of the University of Heidelberg. The two study arms are defined by treatment with arm A (protons at 66 Gy in 20 fractions) and arm B (carbon ions at 66Gy in 20 fractions). Before trial initiation, ethical consent was obtained from the ethics committee of the University of Heidelberg, Germany (Medical Faculty). The number of the ethics committee of Heidelberg (Institutional Review board) is S-298/2011. All patients gave written informed consent before inclusion in the trial.

### Patient selection

Inclusion criteria:

• Histologically confirmed localized prostate cancer with histological classification according to the Gleason score (GS)

• Risk of lymph node involvement < 15% referred to the Yale formula on the basis of T-stage, Gleason score and PSA level (without antihormonal therapy) [4]

$$\begin{array}{ll} \text{Risk}\;\left(\%\right) & =\left(\mathrm{GS}-5\right)\times \left(\text{PSA}/3+1.5\times T\right),\text{with}\;T\\ & =0,1\;\text{and}\;2\;\mathrm{for}\;\mathrm{cT}1c,\mathrm{cT}2a\;\text{and}\;\mathrm{cT}2b/\mathrm{cT}2c \end{array}$$

• PSA level (without antihormonal therapy)

• Age between 40 and 80 years of age

• Karnofsky index ≥ 70%

• Signed written informed consent

Exclusion criteria:

• Previous radiotherapy in the pelvic area

• Distant metastases (stage IV)

• Lymphogenous metastases

• Hip implants or other metal prosthesis at the height of the prostate

• Concurrent participation in other clinical studies, which could influence the results of the respective study

• Active medical implants e.g. pacemaker, defibrillator, which are excluded for ion irradiation

### Work-up

Should a patient meet the trial conditions, information about participation in the study including potential risks and benefits is given to the patient. As soon as written consent is obtained, patients can be included into the IPI trial and all required documentation will be provided by the study center (Studienzentrale Klinische Radiologie, Abt. Strahlentherapie und Radioonkologie, INF 400. 69120 Heidelberg). Before start of treatment an examination including the medical history is taken and the staging examinations where necessary are documented. Before radiation the SpaceOAR (Augmenix, Waltham, MA, USA) is injected at the Department of Urology of the University of Heidelberg. Additionally, a MRI for morphological and functional imaging is conducted. Also the PSA level and QoL (standardized questionnaires EORTC QLQ-C30 and QLQ-PR25) are determined before irradiation. Each patient receives a RT-planning CT-scan in an individually-adjusted precision immobilisation devices (ProStep, ITV, Innsbruck, Austria).

During radiation therapy the patient is supervised by a radiooncologist and clinical symptoms and toxicity (NCI-CTCAE v. 4.02) are documented. Where necessary, a supportive medication is initiated or adapted. The blood count is controlled every week. Morphological and functional MRI examinations are scheduled for midterm of the radiation therapy and during follow up visits (see below). Assessment of NCI -CTC toxicity, QoL as well as functional MRI imaging should take place (Table 1). Follow-up appointments are scheduled.

**Table 1:** Course of the IPI study

**Before radiation**	**Radiation therapy**				**Final examination**	**Radiooncological after treatment**				
	**Week 1**	**Week 2**	**Week 3**	**Week 4**		**+6 weeks**	**+6 months**	**+12 months**	**+18 months**	**+24 months**
• Spacer injection	• Documentation of toxicities and clinical symptoms by the investigator				• Evaluation and documentation of toxicities	• Documentation of PSA level				
• Morphological & functional MRI	• Control of blood counts				• QoL	• Documentation of toxicities				
• Planning CT	• Functional MRI in week 3				• Functional MRI	• Functional MRI				
• PSA level						• QoL (+6 weeks, +6 months and +24 months)				
• QoL										

The post-treatment examinations include measuring the PSA level 6 weeks after the end of treatment and afterwards in three months intervals. It’s important that the PSA level is always determined in the same laboratory to avoid deviations due to differential measurement methods. Additionally, a radiooncological post-treatment examination takes place 6 weeks, 6, 12, 18 and 24 months after the end of treatment including a functional MR imaging. Acute and late toxicity of the irradiation will be assessed on each of the appointments and documented. QoL will be collected at week 6 and after 6 and 24 months. Subsequent to the two years, patient will be after treated according to the current requirements of the guidelines of radiation protection. We will contact the patients at least once a year to ask for PSA-levels and side effects.

### Radiation therapy

#### ***Definition of target volumes and risk organs***

The clinical target volume (CTV) is defined as the prostate and the inferior 2/3 of seminal vesicles plus a margin of 2 mm. The planning target volume (PTV) is defined as the CTV plus 7 mm in lateral direction (beam direction) and 5 mm anterior-posterior as well as in inferior-superior direction. An extra target volume for the rectum (PTV-Rectum) is defined as intersection volume between PTV and rectum. As risk organs are defined rectum, bladder, femoral heads and intestine.

#### ***Definition of the dosage***

95% of the PTV should receive 66 Gy in 20 fractions (5–6 fractions a week) in four weeks. Equivalent doses of the following risk organs (in single doses of 2 Gy) are: for the prostate (α/β = 1.5 Gy) 90.5 Gy, for the bladder (α/β = 4.0 Gy) 80.3 Gy and for the urethra (α/β = 4.5 Gy) 79.2 Gy. The maximal dose for the PTV-Rectum should not exceed 60 Gy. Therefore, the equivalent dose (in a single dose of 2 Gy) is 69.2 Gy (α/β = 3.9 Gy). Other DVH (dose-volume-histogram) constraints are: for the bladder V47 < 30% and V63 < 10%, for the rectum V47 < 30% and V60 < 10%.

### Duration of the study

The recruitment of patients is carried out within two years. The analysis of study results concerning the primary end point (safety and feasibility) will start 6 weeks after the end of treatment of the last patient. Study end point is 24 months after the end of treatment of the last patient. Total study duration is 4 years.

### Evaluation of safety

In radiooncology we differentiate between acute and chronic adverse effects. Acute events are defined to arise within 6 weeks after end of treatment. Data of acute and chronic toxicities are collected during and after treatment on the basis of the NCI-CTC-AE (National Cancer Institute Common Terminology Criteria for Adverse Events) version 4.02 classified into

• Grade 1 = mild; clinical observation; intervention not indicated

• Grade 2 = moderate; minimal, local intervention indicated

• Grade 3 = severe; hospitalization; not immediately life-threatening

• Grade 4 = life-threatening

• Grade 5 = death related to AE

A safe feasibility is defined in this study if no grade 3 toxicity or higher is occurring from beginning of therapy to 6 weeks after the end of treatment without drop out from treatment (see next section). The time till the presentation of a > grade 2 toxicity is another, but secondary, endpoint. All toxicities > grade 2 must be reported the safety board.

### Statistics

The primary objective of the pilot study is to demonstrate the safety and feasibility of the study treatment on the basis of the incidence of grade 3 or higher NCI-CTC AE toxicity and/or a termination of the planned therapy made of any reason. This secure feasibility (SF) is given when from the start of radiation therapy for up to 6 weeks after completion, not any grade 3 or higher toxicity occurred (including toxicity-related death, grade 5) and if the therapy was not stopped due to any other reason, e.g. due to any toxicity (grade 1–4). Taking into account the published very favorable results, the percentage of failure is assumed to be very small and is set to 2.5%, such that the target rate of the SFR was set to 97.5%.

Two co-primary study hypotheses are derived from the questions: a) Is the toxicity of carbon ion irradiation (arm B) non-inferior compared to standard radiation? b) Is the toxicity of the proton irradiation (arm A) non-inferior compared to standard?

Formerly the two hypotheses are indepently test using the nullhypothesis Ho: SFR < 87.5% versus H1: SFR ≥ 97.5%, respectively, for each arm. The decision on the study success is defined for each arm separately. Assuming a type I error of alpha =10% and a power of at least 90% the study needs to recruit per arm n = 41 evaluable patients. This sample size calculation based on the PASS program (Number Cruncher Statistical Systems, October 24 2005, http://www.ncss.com/) and the procedure of Blackwelder (1982) for non-inferiority trials [19]. To replace drop out cases (drop out between consent and the start of treatment or for any other reason), per arm n = 46 patients will be recruited into the study such that a total of N = 92 patients is required. Randomization is performed in blocks of lenths 4 stratified by one dichotomized factor (presence/absence of anti-hormonal therapy during radiation). GS and PSA values will be used for defining post-randomization strata. The analysis of the primary endpoint will be performed by means of one-sample binomial testing. As describing parameters the 97.5% and the 90% confidence limits of the SFR are calculated. NCI-CTC adverse events will be evaluated. PSA-PSF, OS and duration of treatment or study participation are described by means of empirical survival functions. QoL will be evaluated using EORTC QLQ-C-30 guidelines. No formal interim analysis is planned, however, recruitment will be put on hold when the number of failures is larger than 4 in one arm for a discussion of the future of the study in the study team and the Institutional Review Board.

## Discussion

With the conducted study we want to gain basis data of hypofractionated irradiation of the prostate with both, carbon ions and protons in terms of controlled clinical study. Referring to these experiences a confirmatory randomized study comparing carbon ion and proton irradiation will be planned. Hypofractionated carbon ion irradiation is considered as therapy standard since data from Japan are existing, however, not conducted with the raster scan technique. These data serve as historical controls to plan and to evaluate this pilot study regarding safety and feasibility of both planned study arms. Primary endpoint is the evidence of the safety and feasibility of the study treatment on the basis of incidence grade III and IV NCI-CTC-AE (v. 4.02) toxicity and/or the dropout of planned therapy due to any reason.

Both study treatments (arm A: protons; arm B: carbon ions) will be tested separately, but randomized, in a non-inferiority trial for the primary endpoint toxicity since the efficacy of both radiotherapeutical approaches is comparable. The non-inferiority/inferiority is quantified separately by means of the historical control. The question which arm has the advantageous toxicity is evaluated exploratively to plan consecutively a confirmatory study for a full evaluation of efficacy and feasibility of hypofractionated irradiation of the prostate with protons and carbon ions in prostate cancer patients in an active beam technique.

For each of both arms the same non-inferior level of toxicity is chosen and with a width of 10% fits the purpose of a pilot study similarly as the chosen statistical type I error probability of 10%. The calculation of the number of cases occurs on the basis of the both non-inferior questions in comparison to the standard calibrated at the toxicity rate of maximal 2.5% from the current standard (supported by the Japanese results with no reported toxicity).

Randomization will guarantee comparability of both study arms by a balanced patient population in both groups but is not intended for a confirmatory comparison of both arms. Parallel group comparisons are conducted as secondary comparisons.

Aim of the pilot study is the evaluation of the safety and feasibility of hypofractionated irradiation of the prostate with protons and carbon ions in prostate cancer patients in an active beam technique. Additionally, the results of the carbon ion irradiation of the Japanese study are compared in terms of toxicity. A toxicity analysis of the same fractionation scheme with protons will be opposed to the results of the carbon ion irradiation. PSA-PFS, OS and QoL are secondary outcome measures.

## Competing interests

The authors declare that they have no competing interests.

## Authors’ contributions

GH, JD and KH planned and co-ordinate the study. GH, GH, LE, MU, SK, MR, HPS, BH, JD and KH are conducting the study. GH drafted the manuscript. GH, MU, SK, JD and KH are responsible for the patient recruitment. GH, MU, SK, JD and KH perform planning and radiation therapy. GH and BH are responsible for spacer gel application. MR and HPS are responsible for functional MR imaging. KH and LE are responsible for the statistics. All authors read and approved the final manuscript.

## Pre-publication history

The pre-publication history for this paper can be accessed here:

http://www.biomedcentral.com/1471-2407/14/202/prepub
